# Principal Causes of Constitutional Derangement in First Dentition

**Published:** 1881-12

**Authors:** J. Hardman

**Affiliations:** Muscatine, Iowa.


### ARTICLE III.

### *Principal Cause of Constitutional Derangement in First Dentition.*

BY J. HARDMAN, D. D. S., MUSCATINE, IOWA.

This subject has received the attention and investigation of eminent medical men for centuries, and many are the views from the pens of learning in regard to the laws governing and circumstances attending this process at this critical period of the child's life.

The mystery enveloping this subject of first dentition has been a source of great aggravation to the enquiring mind, as exhibited in the great extremes of its effects. At one time the beautiful and expectant pearl has come so normally and so gracefully that the tranquility and health of the little pet has not in the least been disturbed, when again, its advance and eruption is announced by symptoms of suffering and disaster that not only excites anxiety among parents and friends, but alas! too often ends the life of the little sufferer.

Many are the affections that difficult dentition does often induce. Congestions, inflammations, with their attending consequences, attacking any of the vital organisms, producing cholera infantum, dysentery, meningitis, convulsions, etc., etc., in all their direst severity. Able authors have, in our text books, advanced theories to account for this wonderful phenomenon. Have given what they regard as the cause of such extensive and severe effects arising from apparently so insignificant a change of structure. And we read and re-read, weigh and consider, and we find ourself not satisfied with the arguments advanced and many of the conclusions arrived at.

We ask ourself if the physio-vital forces engaged in building up the tooth, cell by cell, and at the same time cell by cell is dissolved and carried away from the surrounding alveolus to fully prepare and maintain ample space without creating the least visible derangement, not only in the immediate parts, but also in the general system, what is it that can so disturb the vital harmony at the time the tooth is just about erupting, and in many cases partially through ?

To supply somewhat of a plausible reply to this perplexing question, this paper has its humble aim, establishment, if possible, a more rational and effective mode of treatment. Permit us, then, while we ask your respectful attention, to first glance briefly at the usually accepted theory ; secondly, present what we regard the true theory, and thirdly, the rational local treatment, as indicated from this standpoint.

We will glance at the anatomical structure, and the physiology of the trigemini, and its co-workers, the sympatheticus major.

The fifth pair of nerves arises low in the base of the brain, and with faciculi or filaments of origin descending down via the pons-varola and medulla-oblongata, making it a spinal as well as an encephalic nerve, bearing in mind that it mostly supplies with its branches all the important parts of the mouth. The great sympathetic nerve, in connection with the trigemini, furnishes extensive influence by its numerous ganglia plexus, anastimosis, etc. This latter nerve is, as it were, the connecting bond of the entire system of nerves—exerting and transmitting wonderful effect, both of voluntary and of involuntary force. This nerve as it descends sends off, externally and internally, branches to every portion of the body, contributing not only to sensibility and motion, but also to special function in most of the vital organs. These, then, the trigemini and the great sympathetic, are not only anatomically connected, but exhibit greatly increased sympathetic influence over the general system.

It is, therefore, when we contemplate this universal and intimate connection of the nervous system, that we to some degree, can comprehend some of the mysterious phenomena where a local irritant in one part or organ may establish the most marked effects in another part or organ. Thus it has been observed where pain in the knee was the result of disease in the hip-joint; where, in the adult the dental so frequently meets, in neuralgia, the seat of pain and distress quite remote from the seat of disease. So in the infant, irritation in the alveoli through the media of impression made upon and through these great nerves, the stomach, bowels, liver, kidneys, lungs, brain, etc., are often the parts upon which the disastrous force is centred, thus causing impaired digestion, diarrhœa, spasms, coughs, convulsions, cephalic congestions and death.

That these dire effects occur from first dentition has long been observed; that the subject is intimately connected with the calling of the Dental practitioner is quite obvious. It is, then, a question of vital importance to the dentist to know the true cause of constitutional derangement in first dentition.

Dr. Dellabarre thought it arises from a defection in the contraction of the investing dentinal capsule, while the lamented Dr. Harris more philosophically thought it may depend upon the pulp being pressed upon; this being caused by the unyielding gums after the osseous opening of the socket has relieved the point of the tooth. The former of these is a vain theory that has no commendable quality; the latter more plausible, but certainly defective in ascribing the pressure upon the nerve as arising from the resistance offered by the overlying gums or soft parts. Drs. Meig, Wood, Bond, Condie, Dungleson, and a host of others, impute, also, to the resistance offered by the gums as the seat and origin of the mischief. We wish, notwithstanding this great weight of talent before us, to be understood as placing no estimate upon the theory that the resistance *from the gums* contribute in any comparative degree towards causing the constitutional trouble. But we do believe most confidently the cause to arise from pressure upon the large and very vital pulp. The question at issue as mostly claiming our attention is, from whence comes this irritating pressure? And why the terrible effects from it?

We maintain that the resistance offered by the gums is but of small moment. That the true cause arises from pressure upon the nerve or pulp is certain, and that this pressure comes from an indirect way, namely, by the growth of the lower or root part of the tooth being too fast, so to speak, when compared to the removal of the osseous covering over its crown, thus holding it back, and it itself thereby pressing unduly and irritating the *pulp*.

We will here, then, in order the more fully to present our views, note what may be regarded as two axioms, viz:

1. "Normal pressure promotes absorption of osseous tissure."

2. "Too much, or undue pressure, will, by inducing irritation, arrest absorption."

It has long since been admitted, and backed by respectable proof, that a given amount of pressure being exerted, will by healthy physiological action, stimulate the removal of osseous structure. And of this principle the dentist takes advantage in the treatment of irregular teeth, even in the mature jaw. But when pressure becomes excessive, instead of a stimulation to activity, the normal function of the membrane compressed is by impediment of circulation, etc., irritated and inflamed, impeding, and in many cases totally arresting action, save that proper to pathological laws—namely, disease.

When we consider that the growth of the deciduous tooth is mainly by deposits of cell by cell, and the filling in of lime salts, particle by particle, from the crown toward the apex; and, further, that the pulp or nerve at this time forms a large portion of the contents of the dentinal capsule, and is of a highly vital organization; and still further, that the normal growth in this portion of the tooth does produce an upward pressure upon and against the capsular covering of the tooth, or lining of the bone; and that through this influence the osseous structure is dissolved and carried away. This process is usually normal before the first opening occurs in the bony casement, as there is seldom evidence of trouble up to this time.

It should be borne in mind that the greatest tendency to constitutional disturbance arises from the eruption of the cuspid teeth. These being the most pointed in form, we might readily be induced to think they should be the most favorable to spontaneous eruption. It is, however, reasonable to conclude from observing the effects, and weighing the real principles of action, that this conical form is no advantage, but instead thereof, the reverse. The point may be through the osseous casement, and in some instances also through the soft coverings of the alveolus, yet the acme of trouble not reached. We shall endeavor here to show the bearing that the process of eruption in this peculiar form of teeth has upon this subject. We maintained that this phenomenon is owing to undue *pressure upon the pulp*, and not upon the tissues in advance of the cutting crown surface. This, then, is our theory, taking a cuspid as a case for illustration. The point is readily felt by the lancet in the hand of the surgeon, but his crucial incissions in the soft tissues have but little effect upon the attending constitutional symptoms. And why? To answer, let us seek from whence this undue pressure.

The point having penetrated the bony surface, a large amount of resistance proper from the growing pressure from below is taken off, hence allowing a greatly increased force being exerted upón the borders of the socket opening, and this force or pressure being too great is an irritant upon the portion of the capsule surrounding this opening, creating inflammation in it, and thus arresting absorption

—holding the tooth, in other words immovably fixed. While thus stationary, the growth of the tooth below still going on, must, it is obvious, in its effect produce, indirectly, pressure upon the *pulp*. And this is the whole mystery, undue pressure, augmented by a portion of resisting surface already removed from over the tooth, and by irritation and inflammation, arresting absorption, fixing the tooth in the socket, while the steadily increasing and elongating tooth indirectly but surely producing the pernicious pressure upon the pulp ; and this in turn becoming by this pressure irritated and inflamed, producing in remote organs, through the intimate nervous union before referred to, the extensive sympathetic disturbance so often met.

It would seem from this illustration that we regard this abnormal change as much more liable in the cuspid teeth, as taking place at this critical time, and that the other teeth are to some extent exempt. This we do think is very much the case, and account for it in this way. These conical teeth have their largest diameter near the middle or base of the crown, thus at the time of penetration of the alveolus by the point and the consequent removal of an equivalent amount of resistance, a much larger surface of the capsule is left to receive the augmented and irritating pressure. Whereas in the more perpendicular crowns, with larger surface, being at once uncovered, leaves but little surface of membrane exposed to this over-pressure and consequent irritation. And we appeal to the experienced practitioner if it is not usually the cuspidati in which the main constitutional disturbance is met.

From the drift of this description it will be noticed that we have dwelt upon the finding and fixing of the true local cause, and its pathology ; but leaving the mysterious philosophy of sympathetic action and phenomena mainly unnoticed. It will, however, be borne in mind that we aimed to point out the cause of, and *not* the effects of this constitutional derangement. We do, however, regard it as befitting and right to close with a word as to the mode of local treatment in first and difficult dentition.

It follows from this view that we have taken that the condition of the soft parts over the tooth is of small moment, save in diagnosis; but that the point to refer our attack in treatment is to relieve the tooth from its strangulation in the bony casement, and thus materially and effectually relieve the pulp from the undue pressure. To do this we would suggest, that with a spear-shaped chisel, or stiff lancet blade the portion of alveolus overlying and surrounding the impinged portion of the tooth be heroically broken up.

Anything short of this will *not* meet the demands of the case. The only plausible good the mere incising of the soft parts may have must be in the reduction of engorgement of the gums through the loss of blood, which must, of course, amount to but little. As testimony of the value of this radical practice, we can say that we have seen, in cases coming under our immediate observation and care, the very best results. But we would insist here that to be effective the surgeon should not hesitate to move or break up the bony casement freely down or nearly to the largest diameter of the crown.—*Dent. Luminary.*

